# Acute Kidney Injury and Intestinal Dysbiosis

**DOI:** 10.3389/fneph.2022.916151

**Published:** 2022-07-07

**Authors:** Jonathan Samuel Chávez-Iñiguez, Luz Yareli Villegas-Gutiérrez, Alejandro Martínez Gallardo-González

**Affiliations:** ^1^ Nephrology Service, Hospital Civil de Guadalajara Fray Antonio Alcalde, Guadalajara, Mexico; ^2^ University Center for Health Sciences, University of Guadalajara, Guadalajara, Mexico; ^3^ Nephrology Service, Regional General Hospital 46, Mexican Social Security Institute, Guadalajara, Mexico

**Keywords:** AKI, acute kidney injury, dysbiosis, intestinal microbiota, inflammation, prebiotics, probiotic

## Abstract

Within the multiple communication pathways of the intestine-kidney axis, one of the most important pathways is the interaction between the commensals of the intestinal microbiome, through the production of short-chain fatty acids, and the segments of the nephron. These interactions maintain a perfect environmental balance. During AKI, there are negative repercussions in all organs, and the systemic interconnection is related in part to the intense inflammation and the uremic environment that this syndrome generates. For example, in the intestine, the microbiome is severely affected, with a decrease in benign bacteria that promote anti-inflammatory effects and an increase in negative, pro-inflammatory bacteria. This scenario of intestinal dysbiosis widens the inflammatory loop that favors worsening kidney function and the probability of dying. It is possible that the manipulation of the intestinal microbiome with probiotics, prebiotics and symbiotics is a reasonable therapeutic goal for AKI.

## Introduction

The intestinal microbiome is made up of millions of bacteria, viruses, fungi and protozoa that interact in health and disease processes. This dynamic and symbiotic relationship modifies the physiological responses of practically any organ and system of our body (1). Studies such as the Human Microbiome Project (HMP) and the Metagenomics of the Human Intestinal Tract (Meta-HIT) have shown how complex the microbiome is. These studies have also presented ample information regarding the discovery of this fascinating interaction, which has coexisted during evolution of the human being (2, 3).

The intestinal microbiota is made up of 100 trillion microorganisms that can be divided into 500 to 1,000 species of bacteria that are specific to each individual. However, there are certain similarities between people who live in the same communities and between families and those who share aspects of daily life, such as diet. These bacteria change according to physiological needs and aging (4). They are cataloged into groups of Bacteroidetes, Firmicutes, and Actinobacteria (5), which represent >90% of all species.

In nonpathological states, intestinal microorganisms (Firmicutes, Bacteroidetes, Actinobacteria, Verrucomicrobia, Proteobacteria) metabolize the components of the diet through proteases, galactosidases, hexosaminidases, tryptophanases and other enzymes (6). Carbohydrates are subjected to a fermentation process and are used as a source of energy. They contribute to the synthesis of short-chain fatty acids (SCFAs), such as acetate, propionate and butyrate (7). These products have a profound impact on the cells of the renal tubule and on the immune system (6). In addition to being a source of energy, they also serve as signaling molecules (7).

Four SCFA receptors have been described in the kidney (Gpr41, Gpr43, Gpr109a, and Olfr78). Gpr41 and Gpr43 are the most studied, they have important physiological roles in the metabolism of renal cells, renal arteries and the immune system, respectively. Olfr78 receptors that are more expressed in afferent renal arteriole and in large renal vessels, promoting hemodynamic changes. Finally, Gpr109a, is the least studied and its physiological effects are not yet known. All of them have the potential to promote cell proliferation, apoptosis, and histone inhibition (7).

During pathological processes, such as inflammation or chronic diseases, the microbiome suffers alterations in the quality and quantity of its components. The bacteria considered benign decrease, and the pathological bacteria trend to proliferate. This generates dysbiosis, which is an event that has been fully identified in patients with impaired renal function (8).

Acute kidney injury (AKI) is a condition in which kidney function deteriorates rapidly; it is a very common syndrome that occurs in 22% of all hospitalized patients (9). It carries a high mortality rate of approximately 23%, and in severe cases, according to the KDIGO classification (10), it can reach a mortality of up to 45%. The clinical outcomes are even worse in emerging countries (11). By its nature, AKI is a highly inflammatory pathology (12) with systemic involvement that affects all organs with profound repercussions on the functioning of all of them. The most common etiology of AKI is sepsis; sepsis increases the risk of developing AKI by up to 210% (13). Compared to other etiologies, AKI associated with sepsis has a poor clinical course, as it has a mortality rate of 49% in the hospital, 45% during intensive care, 36% at 28 days, and 64% at 3 months (14).

There are no specific treatments for AKI, and to date, only its complications have been treated through kidney replacement therapy. However, this treatment does not radically change the trajectory of these patients. The dire need for a treatment for AKI has been suggested as a priority research area by expert consensus (15). To date, various therapeutic targets have failed to improve renal function and mortality in these patients (16–21).

The relationship between intestinal dysbiosis and AKI is bidirectional, that is, dysbiosis generates AKI plausibly by inflammation and AKI promotes and worsens dysbiosis.

### Intestinal Dysbiosis and AKI

Among the many hypotheses about the mechanisms of injury in AKI, the one related to intense systemic inflammation is the best known. AKI directly or indirectly alters the composition of the intestinal microbiome. Then, the permeability of the intestine is dramatically altered by edema, hypoperfusion and ischemia, leading to a “leaky gut” that activates the immune response by bacterial translocation (1). This response generates and amplifies systemic inflammation. During this process, the gut microbiota interacts with sepsis and AKI independently.

In a symbiotic (normal) intestinal environment in which commensal bacteria proliferate and generate SCFAs, the intestinal integrity is strong and predominantly anti-inflammatory. However, in a uremic environment due to AKI or chronic kidney disease (CKD), the intestinal integrity is degenerated. Thus, intestinal dysbiosis occurs with the increased production of uremic toxins, resulting in a decrease in saccharolytic bacteria and proliferation of proteolytic bacteria. There are also reports on bacterial translocation (elevated during AKI) as a consequence of uremia (22). Urea is broken down in the intestine by bacteria that have the enzyme urease and is converted into ammonium and ammonium hydroxide. At the epithelial junctions of the intestine, these products increase transluminal flow and the translocation of bacteria from the intestine to the systemic circulation. Thus, the activation of Th17, Th1 cells, B cells and macrophages, a proinflammatory environment are promoted (6). These events of dysbiosis and increased inflammation are believed to be synergistic between AKI and sepsis, and are referred to as gut-kidney interactions.

In the renal tubule, the microbiota communicates with the ligands FFA3, FFA2 and OR51E2 *via* SCFAs. This interaction produces a change in cell metabolism (7). It has been observed that the systemic inflammatory response decreases up to 10,000 times the amount of intestinal bacteria considered beneficial, such as *Bifidobacterium* and *Lactobacillus*. In contrast, it increases up to 100 times the number of pathogenic bacteria, such as *Staphylococcus* (23). This translates into a decrease in the generation of SCFAs, assessed by an increase in the fecal pH. The fecal pH of patients with inflammation can reach up to 7.4, while a normal fecal pH is 6.6 in healthy patients (reflecting dysbiosis) (24).

It has also been reported that the number of obligate and facultative anaerobes is related to mortality in critically ill patients (24). Along these lines, in a cohort of 82 soldiers who had suffered AKI during combat, it was established through metabolomics measurement (84 metabolites) that the most important determinants for the severity of their AKI and the requirement for kidney replacement therapy are directly related to the intestinal microbiome by the expression of indoxyl sulfate, indole-3-acetate, hippurate and phenylacetylglycine (25).

### Experimental Evidence in Animal Models on the Interaction and Modulation of Dysbiosis and AKI

The evidence obtained through experimental studies that justifies the hypothesis of the interaction between intestinal microbiome, dysbiosis and AKI is extensive (**Table 1**).

**Table 1:** Experimental studies on the use of SCFAs in AKI models.

Study	Objective	Model	Population	Method	Results	Conclusions
**Andrade-Oliveira V et al.** (26)	Whether treatment with SCFAs has a protective effect in mice with IRI and whether this protection involves direct modulation of inflammatory processes and/or reduction of oxidative stress.	IRI	*In vivo*: mices *In vitro*: renal epithelial cells	Acetate, propionate, butyrate 200 mg/kg, IP injection two doses, 30 minutes before ischemia and at the time of reperfusion. *B. longum* o *B. adolescentis* (10^8^) by gavage for 10 days Acetate (25 mM), Propionate (12 mM), Butyrate (3.2 mM) treat renal epithelial cell line	HIF-1a translocation to the nucleus was decreased and there were lower lactate levels.Increase autophagy and tubular proliferating cells. Inhibit NF-kB activation, apoptosis, and ROS productionSCFA treatment reduced the expression of the costimulatory molecules CD80 and CD40 in bone marrow DCs Treatment of APCs with LPS plus SCFAs was sufficient to reduce the proliferation of CD8 and CD4 cells	Treatment with acetate preserved renal structure, which was reflected by an improvement in the tubular epithelial cell necrosis score.Modulation by inhibition of local and systemic inflammation, oxidative stress, cell infiltration and activation, apoptosis, autophagy and chromatin modification.
**Park J, et al.** (27)	The impact of elevated SCFAs levels on T cells and inflammatory tissues in mice.	Immune system	*In vivo*: mices *In vitro*: renal T cells	Sodium acetate (100, 150, 200 mM), sodium propionate (200 mM) or sodium butyrate (200 mM) or rapamycin (25 mg/ml) and/or vancomycin (0.5 g/l) Sodium acetate (10 mM), sodium propionate (1 mM), sodium Butyrate (0.5 mM) and/or rapamycin (25 nM)	Increase in Th17 y Th1 cells. Inflammation and ureteral hyperplasia occur Inflammation induced by high levels of SCFAs can be suppressed by rapamycin.	T-cell mediated urethritis leads to renal hydronephrosis. It depends on mTOR and the deficiency of GPR41 or GPR43 did not affect the development
**Sun X et al.** (28)	The effects of sodium butyrate on gentamicin-induced nephrotoxicity	Gentamicin-induced AKI	*In vivo*: rats	Sodium butyrate (50, 100, 200 mg/kg) by injection given 30 minutes before the injection of gentamicin.	Sodium butyrate is a histone deacetylase inhibitor that enhances the activity of antioxidant enzymes and the expression of prohibitin protein.	Sodium butyrate significantly attenuated gentamicin-induced AKI by increasing superoxide dismutase, catalase, prohibitin and reduced glutathione.
**Machado RA, et al.** (29)	The effect of sodium butyrate in rats with contrast-associated nephropathy	Contrast-associated AKI	*In vivo*: Wistar rats	The contrast was administered for 6 days. Sodium butyrate (500 mg/kg) was administered by intravenous injection.	Decreased lipid peroxidation levels but not protein oxidation and IL-6 levels. Decreased NF-kB translocation and tubular damage.	The protective effect is by inhibition of NF-kB expression.
**Jang HR, et al.** (30)	Whether differences in perinatal microbial status modify kidney after IRI	IRI	*In vivo*: mices	Basal intrarenal resident lymphocytes and cytokine levels were compared. The renal pedicles were clamped for 35 minutes.	Normal kidneys from germ-free mice exhibited more NK cells and lower levels of IL-4. Structural injury and renal impairment were more severe in germ-free mice.	Microbial stimuli influence phenotype and cytokine expression and modulate IRI outcome.
**Yusuke Nakade et al (** 31 **),**	Whether the gut microbiota protects tubular injury in AKI, the effect of AKI on the microbiota, and whether D-serine reduces tubular injury	IRI	*In vivo*: mices with IRI *In vivo*: mices with or without fecal transplant	Sequencing analysis of the 16S rRNA gene in mouse feces at days 0, 2 and 10 after IRI. 20 mM of D or L-serine were administered to the mice.	Decrease of *Bifidobacterium* y *TM7* and increased of *Lactobacillus*, *Clostridium* y *Ruminococcus* in IRI At day 2, AKI was worse in germ-free mice and by day 10, the damage progressed to ATN. Decreased expression of HAV cellular receptor 1 mRNA after fecal transplantation. D-serine suppresses damage and promotes hypoxia-mediated TEC proliferations at values of 1-100 uM D-serine.	The AKI score was higher in mice treated with antibiotics. D-serine levels reflect renal function in patients with AKI.
**Diba Emal, et al.** (32)	The importance of the microbiome in the development of kidney disease.	IRI	*In vivo*: mices 8-12 weeks-old	Microbiota depletion occurred with antibiotics (ampicillin 1 g/l, metronidazole 2 g/l, neomycin 1 g/l and vancomycin 0.5 g/l).fecal. IRI was induced with unilateral or bilateral clamping of the renal pedicles for 25 minutes.	Decreased kidney damage, dysfunction, and injury to distant organs. Lower levels of F4/80 and CX3CL1 y CCL2 receptors in macrophages and monocytes.	Gut microbiota depletions protects by slowing the maturation of F4/80+ renal residents and monocytes.
**Nadezda V. Andrianova, et al.** (33)	Whether the intestinal microbiota can affect the severity of AKI and identify specific bacterial taxa that have positive or negative roles in the progression of kidney damage.	IRI	*In vivo*: rats	Creatinine, urea, and number of AA and acylcarnitines were measured. Left kidney ischemia was induced for 40 minutes followed by reperfusion.	Tyrosine, tryptophan, and proline decreased by 60-70%. Malonylcarnitine increased 7-fold. The logarithmic relationship between *Rothia* and *Streptococcus* is the best predictor of the creatinine value (p= 0.0014, adjusted R2 = 0.55).	Acylcarnitines have a positive correlation with creatinine, while AA had a strong negative correlation with coefficients less than 0.5

AA, Amino acids; AKI, Acute Kidney Injury; ATN, acute tubular necrosis; DCs, Dendritic cells; GPR, G protein coupled receptors; HAV, hepatitis A virus; HIF-1a, Hypoxia-inducible-factor 1 alpha; IRI, Ischemia-reperfusion injury; mTOR, Mammalian target of rapamycin; NF-kB: nuclear factor kappa B; NK, Natural Killers; ROS, Reactive Oxygen Species; SCFAs, Short-chain fatty acids; TEC, tubular epithelial cells.

In a mouse model of renal ischemia/reperfusion, the proliferation of bacteria such as *Rothia* sp., and *Staphylococcus* sp. was consistent with the severity of AKI and correlated linearly with the elevation in creatinine and urea (33). In rats with microbiota depletion prior to an episode of AKI due to ischemia reperfusion, there was a notable decrease in markers of inflammation and tubular damage after the event. In mice depleted of intestinal bacteria with the use of antibiotics, the percentage of expression of the classic inflammatory markers and renal macrophages did not change despite a reduction in chemokine receptors. Likewise, in mice without intestinal microbiota, the benefit that they had presented was reversed once a fecal transplant was performed (32).

In another experimental model, AKI was induced in rats by urosepsis *via* E. coli inoculation. In this model, the administration of probiotics (*Lactobacillus acidophilus* and *Bifidobacterium*) one month before and two months after renal insult significantly attenuated the histological evidence of kidney injury (34).

When comparing sham rats with those that underwent cecal ligation, the administration of *Lactobacillus rhamnosus GG* and *Bifidobacterium longum* for 7 days was associated with a 40% decrease in the risk of dying (35).

Another experimental study administered SCFAs to rats after AKI in an ischemia/reperfusion model and found improvement in renal function and tissue damage. They observed a decrease in all inflammatory factors, such as TNF alpha, IL-6, IL-1b and MCP-1 (26).

Another study found that the protection conferred by acetate administration was associated with a decrease in the production of reactive oxygen species, a local decrease in cytokines and chemokines, lower levels of receptor messenger RNA, toll-type 4 and its ligand (biglycan), and lower activation of NF-kB.

Similarly, before the induction of AKI by cisplatin, the administration of lactic acid bacteria (*Lactobacillus salivarius*) maintained lower levels of creatinine, urea, inflammatory markers, and tissue damage scores (36); additionally, the integrity of the intestinal wall barrier was maintained.

## Human Clinical Evidence of Intestinal Dysbiosis in AKI

Studies on intestinal dysbiosis in humans with AKI are very scarce, but some findings suggest that the biology of humans is similar to that of the experimental studies. It may be that the influence of the microbiome on AKI is based on the “Hygiene hypothesis”, which establishes how the microbiome (physiological) induces less inflammation (37). In addition, the same uremia generated with AKI promotes dysbiosis, and urea degrades the intestinal mucosa, which also facilitates this event. As an example, 13 patients with AKI demonstrated increases in the production of D-amino acids (D-serine) produced by SCFAs (abundant in symbiosis). This is an event that could represent a kidney protection mechanism by intestinal bacteria before kidney insult (31). Patients with AKI and sepsis receive broad-spectrum antibiotics, which have a profound impact on the gut microbiota by changing its conformation (38).

In CKD, the quantity of uremic toxins derived from the colon, such as indoxyl sulfate (IS), increases; however, it seems that this situation also occurs in AKI.

In a prospective cohort of 262 patients with AKI, an association was identified between high levels of IS and mortality, increasing this outcome threefold (39). In another cohort of 194 patients in an intensive care unit, improvement in the RIFLE score in AKI was observed in those who had a decrease in these colon-derived toxins (40). Multiple studies have been conducted to assess the effect of symbiosis on IS and p-cresol-sulfate concentrations and other markers in predialysis CKD patients.

## Therapeutic Advances

**Figure 1:**
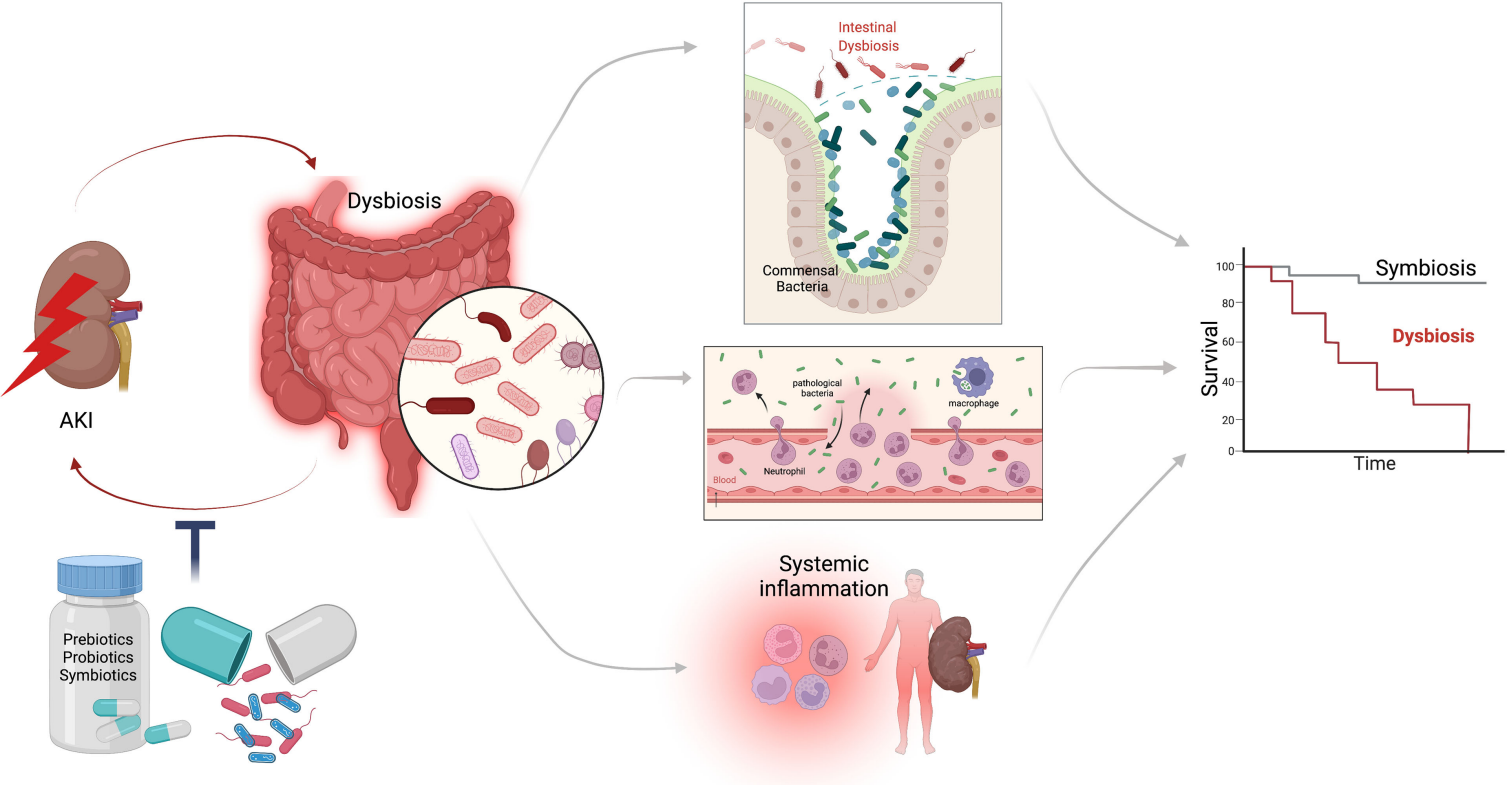
Interaction between AKI and intestinal dysbiosis. During AKI multiple mechanisms lead to alterations in the conformation of the intestinal microbiome, promoting the proliferation of pathogenic bacteria and the decrease of beneficial bacteria, an event known as dysbiosis; which generates rupture of the intestinal barrier, bacterial translocation, and systemic inflammation; further worsening kidney function, preventing its recovery. Dysbiosis-AKI loop has been associated with worse survival. It is possible that the manipulation of the intestinal microbiome with prebiotics, probiotics and symbiotics can attenuate dysbiosis and thus improve the clinical outcomes in AKI patients.

Probiotics are live microorganisms that, when administered in the appropriate amounts, offer benefits to the host. The combination of both prebiotics and probiotics is called symbiotic, and they are usually administered in food supplements (41, 42). Until now, the experimental studies are encouraging since they have been favorable in reducing systemic inflammation, improving renal function parameters and even histological damage **(** **Figure 1**). The information derived from studies in patients with CKD supports the rationale for testing this therapeutic target in the context of AKI, a clinical entity that carries high morbidity and mortality. Recently, Rydzewska-Rosołowska et al. (1) conducted a systematic review identifying only 5 human studies (three prospective and two retrospective); however, up until the time of this review, there were no clinical trials with the use of probiotics in AKI (24, 43).

## Conclusions and Perspective

The intestinal dysbiosis that is generated during AKI deteriorates the clinical course and amplifies kidney damage through multiple pathways. Dysbiosis has systemic involvement, and inflammation seems to be the most involved pathway of damage. Modifying clinical outcomes in AKI through modulation of the microbiome is biologically plausible, and we could manipulate that environment. Probiotics are a good option; probiotics are live microorganisms that, when administered in the appropriate amounts, offer benefits to the host. Furthermore, despite years of clinical research, no treatment has been discovered that truly modifies the clinical course of AKI. We are running a randomized, double-blinded, placebo-controlled clinical trial (Clinical Trails registry: NCT03877081), in which we will evaluate the effect of the administration of prebiotics, probiotics and symbiotics for 7 days in patients with AKI induced by sepsis. We are waiting for their results, which may elucidate the fascinating association between the gut microbiota and AKI.

## Author Contributions

JSCI, LYVG and AMGG contribute equally to elaborate this mini-review. All authors contributed to the article and approved the submitted version.

## Conflict of Interest

The authors declare that the research was conducted in the absence of any commercial or financial relationships that could be construed as a potential conflict of interest.

## Publisher’s Note

All claims expressed in this article are solely those of the authors and do not necessarily represent those of their affiliated organizations, or those of the publisher, the editors and the reviewers. Any product that may be evaluated in this article, or claim that may be made by its manufacturer, is not guaranteed or endorsed by the publisher.
